# Addition of four doses of rituximab to standard induction chemotherapy in adult patients with precursor B-cell acute lymphoblastic leukaemia (UKALL14): a phase 3, multicentre, randomised controlled trial

**DOI:** 10.1016/S2352-3026(22)00038-2

**Published:** 2022-03-28

**Authors:** David I Marks, Amy A Kirkwood, Clare J Rowntree, Melanie Aguiar, Katharine E Bailey, Brendan Beaton, Paul Cahalin, Anna Z Castleton, Laura Clifton-Hadley, Mhairi Copland, Anthony H Goldstone, Richard Kelly, Emma Lawrie, SooWah Lee, Andrew K McMillan, Mary Frances McMullin, Tobias F Menne, Rachel J Mitchell, Anthony V Moorman, Bela Patel, Pip Patrick, Paul Smith, David Taussig, Deborah Yallop, Krisztina Zuborne Alapi, Adele K Fielding

**Affiliations:** a United Bristol Healthcare Trust, Bristol, UK; b CR UK and UCL Cancer Trial Centre, Cancer Institute, University College London, London, UK; c Cardiff and Vale University Health Board, Cardiff, UK; d University College London Cancer Institute, London, UK; e Blackpool Teaching Hospitals NHS Foundation Trust, Blackpool, UK; f The Christie NHS Foundation Trust, Manchester, UK; g Paul O'Gorman Leukaemia Research Centre, College of Medical Veterinary and Life Sciences, University of Glasgow, Glasgow, UK; h St James's University Hospital, Leeds, UK; i Centre for Clinical Haematology, Nottingham City Hospital, Nottingham, UK; j Haematology, Queen's University, Belfast, UK; k Newcastle Upon Tyne Hospitals NHS Foundation Trust, Newcastle upon Tyne, UK; l Leukaemia Research Cytogenetics Group, Translational and Clinical Research Institute, Newcastle University, Newcastle, UK; m Barts Cancer Institute, The London School of Medicine, Queen Mary University of London, London, UK; n Haemato-Oncology Section, Royal Marsden Hospital, Sutton, UK; o King's College Hospital NHS Foundation Trust, London, UK

## Abstract

**Background:**

Treatment for adults with acute lymphoblastic leukaemia requires improvement. UKALL14 was a UK National Cancer Research Institute Adult ALL group study that aimed to determine the benefit of adding the anti-CD20 monoclonal antibody, rituximab, to the therapy of adults with de novo B-precursor acute lymphoblastic leukaemia.

**Methods:**

This was an investigator-initiated, phase 3, randomised controlled trial done in all UK National Health Service Centres treating patients with acute lymphoblastic leukaemia (65 centres). Patients were aged 25–65 years with de-novo *BCR-ABL1*-negative acute lymphoblastic leukaemia. Patients with de-novo *BCR-ABL1*-positive acute lymphoblastic leukaemia were eligible if they were aged 19–65 years. Participants were randomly assigned (1:1) to standard-of-care induction therapy or standard-of-care induction therapy plus four doses of intravenous rituximab (375 mg/m^2^ on days 3, 10, 17, and 24). Randomisation used minimisation and was stratified by sex, age, and white blood cell count. No masking was used for patients, clinicians, or staff (including the trial statistician), although the central laboratory analysing minimal residual disease and CD20 was masked to treatment allocation. The primary endpoint was event-free survival in the intention-to-treat population. Safety was assessed in all participants who started trial treatment. This study is registered with ClincialTrials.gov, NCT01085617.

**Findings:**

Between April 19, 2012, and July 10, 2017, 586 patients were randomly assigned to standard of care (n=292) or standard of care plus rituximab (n=294). Nine patients were excluded from the final analysis due to misdiagnosis (standard of care n=4, standard of care plus rituximab n=5). In the standard-of-care group, median age was 45 years (IQR 22–65), 159 (55%) of 292 participants were male, 128 (44%) were female, one (<1%) was intersex, and 143 (59%) of 244 participants had high-risk cytogenetics. In the standard-of-care plus rituximab group, median age was 46 years (IQR 23–65), 159 (55%) of 294 participants were male, 130 (45%) were female, and 140 (60%) of 235 participants had high-risk cytogenetics. After a median follow-up of 53·7 months (IQR 40·3–70·4), 3-year event-free survival was 43·7% (95% CI 37·8–49·5) for standard of care versus 51·4% (45·4–57·1) for standard of care plus rituximab (hazard ratio [HR] 0·85 [95% CI 0·69–1·06]; p=0·14). The most common adverse events were infections and cytopenias, with no difference between the groups in the rates of adverse events. There were 11 (4%) fatal (grade 5) events in induction phases 1 and 2 in the standard-of-care group and 13 (5%) events in the standard-of-care plus rituximab group). 3-year non-relapse mortality was 23·7% (95% CI 19·0–29·4) in the standard-of-care group versus 20·6% (16·2–25·9) in the standard-of-care plus rituximab group (HR 0·88 [95% CI 0·62–1·26]; p=0·49).

**Interpretation:**

Standard of care plus four doses of rituximab did not significantly improve event-free survival over standard of care. Rituximab is beneficial in acute lymphoblastic leukaemia but four doses during induction is likely to be insufficient.

**Funding:**

Cancer Research UK and Blood Cancer UK.

## Introduction

Although outcomes of adult acute lymphoblastic leukaemia have improved in recent years, fewer than 50% of older adults might expect to be cured with first-line combination chemotherapy. Rituximab is a monoclonal antibody targeting cell surface antigen CD20, which is expressed on more than 20% of acute lymphoblastic leukaemia blasts in about 40% of adults with the disease. Rituximab is generally well tolerated and has benefitted patients with a wide range of B-cell malignancies. In a single-arm, single-centre study from the MD Anderson Cancer Centre, which included 282 adolescent and adult patients with *BCR-ABL1*-negative acute lymphoblastic leukaemia, in whom more than 20% of blasts expressed CD20, rituximab was added to hyperfractionated cyclophosphamide, doxorubicin, vincristine, and dexamethasone (hyperCVAD) for a total of 12 doses.^1^ There was an overall survival benefit for the addition of rituximab compared with historical controls (75% *vs* 47%; p=0·003), although older patients did not benefit from rituximab due to a higher rate of death in complete remission. The French GRAALL (Groupe de Recherche sur les Leucémies Aiguës Lymphoblastique) group did a randomised controlled trial of the addition of 16–18 doses of rituximab to standard of care compared with standard of care alone in 209 adults aged 18–59 years with *BCR-ABL1*-negative acute lymphoblastic leukaemia, in whom more than 20% of blasts expressed CD20.^2^ Event-free survival in patients receiving the rituximab chemotherapy regimen was significantly improved compared with the control group (hazard ratio [HR] 0·66 (95% CI 0·45–0·98]; p=0·04), although the difference in overall survival was not significant (HR 0·70 [0·46–1·07]; p=0·10). Among patients in the same study^2^ who received an allogeneic haematopoietic stem-cell transplantation (HSCT), event-free survival and overall survival benefit remained apparent in the rituximab group. There was a non-significant interaction between event-free survival and CD20 expression greater than the median (HR 0·82 [0·47–1·42]) and less than the median (HR 0·52 [0·29–0·93]; p=0·40), but patients with CD20 expression on fewer than 20% blasts did not enter the trial. Of interest, CD20 is upregulated on B-cell acute lymphoblastic leukaemia blast cells by corticosteroids, suggesting that a relationship between benefit from rituximab and baseline CD20^3^ cannot necessarily be anticipated. Additionally, higher levels of cell surface CD20 expression have been identified by some studies as an adverse prognostic biomarker in acute lymphoblastic leukaemia,^4^, ^5^, ^6^, ^7^, ^8^ although several studies do not concur.^9^, ^10^, ^11^ This is an important consideration, because studies that recruited patients with higher CD20 expression might have selected a subset of patients with a worse prognosis.

**Research in context**

**Evidence before this study**

We searched PubMed using the terms rituximab and acute lymphoblastic leukaemia, with no limits on the date of publication and no language restrictions. Although the anti-B-cell monoclonal antibody rituximab brought considerable survival improvements to the outcomes of patients with lymphomas, at the onset of this study the only published data in B-cell acute lymphoblastic leukaemia was a single-arm study that showed benefit over historical controls, in patients with more than 20% of B-cell acute lymphoblastic leukaemia cells expressing CD20. Patients older than 60 years did not benefit due to a higher rate of death in complete remission. A randomised study adding rituximab to the treatment of B-cell acute lymphoblastic leukaemia was initiated by the GRAALL group in France and ran concurrently with our trial. The GRAALL trial enrolled patients with *BCR-ABL1*-negative acute lymphoblastic leukaemia aged 18–59 years with more than 20% of acute lymphoblastic leukaemia cells expressing CD20. On the basis of laboratory studies showing that pretreatment with corticosteroids upregulated CD20 expression on B-cell acute lymphoblastic leukaemia cells and there being no reason to assume benefit would be confined to the subset of patients with *BCR-ABL1*-negative disease, the UK NCRI Adult ALL group designed the UKALL14 trial, in which patients aged 25–65 years with B-cell acute lymphoblastic leukaemia were randomly assigned to standard of care, with or without rituximab, regardless of baseline CD20 expression or *BCR-ABL1* status.

**Added value of this study**

3-year event-free survival was not significantly different between the treatment groups. However, several new findings emerge from this study. We showed that rituximab was safe at all ages and did not engender additional toxicity, even in the large number of patients in whom allogeneic haematopoietic stem-cell transplantation (HSCT) was a subsequent therapy. Dexamethasone upregulated CD20 on primary acute lymphoblastic leukaemia blasts in vivo in six of eight patients tested. We found no interaction with CD20 baseline expression, consistent with upregulation of CD20 by steroids. Treatment effects on event-free survival were generally larger for patients with *BCR-ABL*-positive than *BCR-ABL*-negative disease, with significant difference for bone marrow relapses. The treatment effect was greatest for those who received myeloablative-conditioned allogeneic HSCT, who had a significantly greater event-free survival if treated in the rituximab group. This finding is not readily explained; both relapse risk and non-relapse mortality were reduced by the same magnitude and there was no apparent difference in graft-versus-host disease rates.

**Implications of all the available evidence**

The results from GRAALL and our trial suggest rituximab can be beneficial for adults with B-cell acute lymphoblastic leukaemia. UKALL14 suggests that any benefit is not confined to those with *BCR-ABL1*-negative disease or with CD20 expression on more than 20% of B-cell acute lymphoblastic leukaemia cells. However, the full benefit of rituximab in acute lymphoblastic leukaemia would require administration of rituximab throughout the therapy—eg, 16–18 doses, as used in the GRAALL randomised controlled trial.

We aimed to determine the effect on event-free survival of four doses of rituximab given during initial induction therapy for patients with B-cell acute lymphoblastic leukaemia. We chose to evaluate fewer doses than other studies to avoid potential toxicity in older patients and because we hypothesised that benefit from rituximab would accrue from early administration, generating a deeper molecular response that would be measured as a secondary endpoint. Here, we report the final analysis of the UKALL14 B-cell arm randomisation.

## Methods

### Study design and participants

UKALL14 was a UK-wide, investigator-initiated, phase 3, randomised controlled trial with three randomisations as well as a single-arm question on the role of reduced-intensity transplantations for patients aged 40 years and older.^12^ The trial protocol is in the appendix (pp 25–178). Patients were aged 25–65 years with de-novo *BCR-ABL1*-negative acute lymphoblastic leukaemia. Patients with de-novo *BCR-ABL1*-positive acute lymphoblastic leukaemia were eligible if they were aged 19–65 years as there were no other trial options available in the UK for patients aged 19–24 years with this condition. A steroid prephase of 5–7 days was required and was the only therapy that could commence before formal trial registration. The only exclusions from trial entry were for HIV infection, active hepatitis B infection, pregnancy or lactation**,** blastic transformation of chronic myeloid leukaemia, and mature B-cell leukaemia. There were no exclusions for previous malignancies, poor organ function, laboratory test abnormalities, or poor performance status. Dose adjustments to standard-of-care medications were permitted for organ dysfunction or laboratory test abnormalities, according to the summary of product characteristics.

The trial was managed by the Cancer Research UK and University College London Cancer Trials Centre (UCL CTC) and all UK National Health Service centres treating patients with acute lymphoblastic leukaemia participated (65 centres in total; appendix pp 18–19). Ethical approval was obtained from the London–Fulham Research and Ethics Committee. Written informed consent was obtained in accordance with the Declaration of Helsinki.

After 91 patients had been recruited, the induction protocol was amended on April 24, 2012, due to an unexpectedly high induction death rate.^13^ The induction dose of daunorubicin was halved, the day 4 dose of pegylated asparaginase was removed for patients aged 41 years and older, and all doses of pegylated asparaginase were removed for patients with *BCR-ABL1*-positive disease. 91 additional patients were recruited, and this paper focuses on only the eligible patients recruited after this amendment.

### Randomisation and masking

Patients were randomly assigned (1:1) using a computer-based system at diagnosis to standard of care or standard of care plus rituximab, regardless of *BCR-ABL1* status and irrespective of cell surface CD20 expression, at UCL CTC using minimisation (including a random element of 0·9) and stratified by sex, age (≤40 *vs* >40), and white blood cell count (<30 × 10^9^ cells per L *vs* ≥30 × 10^9^ cells per L). No masking was used for patients, clinicians, or UCL CTC staff including the trial statistician, although the central laboratory analysing minimal residual disease (MRD) and CD20 was masked to treatment allocation.

### Procedures

All patients completed a 5–7 day prephase of oral dexamethasone 6 mg/m^2^ per day followed by two sequential courses of induction therapy (induction phase 1 and 2). Induction phase 1 chemotherapy in the standard-of-care group consisted of intravenous daunorubicin 30 mg/m^2^ and vincristine 1·4 mg/m^2^ (2 mg maximum) on days 1, 8, 15, and 21, oral dexamethasone 10 mg/m^2^ on days 1–4, 8–11, and 15–18, and a single 12·5 mg intrathecal methotrexate dose on day 14. Intravenous pegylated asparaginase 1000 IU/m^2^ was given on day 4 and day 18 to patients aged 40 years and younger, and on day 18 only to those aged 41 and older. Patients with *BCR-ABL1*-positive acute lymphoblastic leukaemia received the same treatment without pegylated asparaginase and with the addition of continuous oral imatinib from day 1, starting at 400 mg and escalating to 600 mg, given daily. For patients in the standard-of-care plus rituximab group, intravenous rituximab was added to standard of care at 375 mg/m^2^ given on days 3, 10, 17, and 24. Antibacterial, antiviral, and antifungal prophylaxis was mandated for all patients, but centres used local policy for choice of agents.

All patients received induction phase 2 (appendix p 20) followed by risk assessment. Standard-risk treatment was with high dose methotrexate, four consolidation courses (which included one delayed intensification) and 2 years of oral maintenance therapy with 3-monthly vincristine and steroid pulses. Participants were assessed as being at high risk if they presented with a white cell count more than 30 × 10^9^ cells per L, were *BCR-ABL1*-positive*,* had *KMT2A/AFF1*, had low hypodiploidy/near triploidy (<40 chromosomes), had complex cytogenetics (more than five abnormalities), had any positive MRD at the end of induction phase 2, or were aged 41 years or older.

High-risk treatment was assigned by age. Those aged 40 years and younger (and patients with standard-risk acute lymphoblastic leukaemia and a sibling donor, at physician and patient choice) were assigned myeloablative allogeneic HSCT. Those aged 41 years and older were assigned reduced-intensity conditioned allogeneic HSCT to be given after a high dose methotrexate intensification. Details of post-induction treatments are shown in the appendix (p 20). Reduced-intensity conditioned allogeneic HSCT in all patients aged 41 years and older was evaluated in a separate group of the trial to examine efficacy and safety of a fludarabine, melphalan, and alemtuzumab regimen, the results of which are reported elsewhere.^12^ Details of UKALL14 treatments are shown in the appendix (p 20). Details of flow cytometric analysis of CD20 are shown in the appendix (p 1). Data on race and ethnicity were not collected.

Complete remission was assessed on bone marrow aspirates in local centres and defined as less than 5% lymphoblasts by conventional light microscopy in the presence of peripheral blood neutrophil count greater than 0·75 × 10^9^ cells per L and platelet count greater than 75 × 10^9^ platelets per L. All patients were expected to have central laboratory quantification of MRD using clonal immunoglobulin or T-cell receptor gene rearrangement quantification (or *BCR-ABL* transcript quantification for *BCR-ABL*-positive acute lymphoblastic leukaemia). MRD was quanitifed according to EuroMRD criteria in the EuroMRD-accredited UK Adult ALL MRD laboratory at UCL Cancer Institute (London, UK).^14^, ^15^ The MRD result at the end of induction phase 1 was not released to the treating centre and did not affect treatment decisions. The induction phase 2 result was supplied to centres as negative, positive (if positive, a quantification was given), positive outside the quantitative range (POQR), or indeterminate (assay did not reach the required minimum assay sensitivity <10^−4^) and formed part of the risk assessment. Where an MRD result was unavailable or classified as either indeterminate or POQR, the result was treated as negative to prevent assignment to allogeneic HSCT due to a non-quantifiable risk. Hence, POQR results were analysed together with negative results. To analyse MRD as a continuous variable, negative results were set to 10^−6^ and POQR as 10^−5^.

The standard Common Terminology Criteria for Adverse Events reporting system (version 4.0) was used for adverse event reporting, with the worst grade of each event recorded during, and up to 30 days after, each treatment phase excluding maintenance. Patients were not removed from the study for adverse events nor for omissions, delays, or dose reductions of standard drugs, but these events were also captured.

### Outcomes

The primary endpoint was event-free survival. Secondary endpoints were: the complete remission rate at the end of induction phase 2, overall survival, cumulative incidence of relapse (including bone marrow and CNS only), non-relapse mortality, MRD at the end of phase 1 induction, and anti-asparaginase antibody formation. The results for anti-asparaginase antibody formation are not yet available and will be reported elsewhere. Additional secondary endpoints that did not relate to this particular trial question and will be reported elsewhere were MRD quantification after allogeneic HSCT, toxicity related to pegaspargase, rates of hypersensitivity, changes to *Erwinia* or withdrawal of asparaginase treatment, acute and chronic graft-versus-host disease (GVHD) rates, and mucositis scores in patients receiving palifermin.

Event-free survival and overall survival times were measured from the date of random assignment until the date of event (relapse [>5% blasts, local assessment] or death for event-free survival or death for overall survival) or until the date last seen (patients without an event).

### Statistical analysis

It was assumed that the 3-year event-free survival in the standard-of-care group was 40% and the trial aimed to show a 12% improvement to 52% with the addition of rituximab (ie, an HR of 0·71). Using a two-sided 5% α, 307 events were needed to give 84% power to show this difference. Assuming a minimum follow-up of 6 months after the last random assignment, 576 patients were needed. We analysed event-free survival and overall survival using Kaplan-Meier survival analysis, with treatment groups and prespecified subgroups (age, sex, white blood cell count, cytogenetic risk group, body-mass index, and extramedullary involvement) compared using Cox regression and the log-rank test. An analysis of the primary endpoint adjusted for baseline stratification factors was also done. Cumulative incidence of relapse and non-relapse mortality times were measured from the date of remission until the date of the event or the date last seen. These were analysed using competing risks analysis by the method of Fine and Gray, with death in remission and relapse treated as competing events (non-bone marrow or non-CNS relapse were also included as competing risks for cumulative incidence of bone marrow and CNS relapse). Landmark analyses were used for the reduced-intensity conditioning and myeloablative conditioning cohorts, with time measured from the date of transplantation. The proportional hazards assumption was tested using the Schoenfeld residuals. For these groups, the cumulative incidence of GVHD was also calculated using competing risks (relapse and death) with time measured from the date of transplantation until the first occurrence of either acute or chronic GVHD. Multivariable analyses including known, prespecified, clinical baseline risk factors (and HSCT for time varying analyses) were also done for all time-to-event endpoints. These analyses were adjusted for the same factors used in the subgroup analyses above (and transplantation as a time varying covariate, where mentioned). These were considered as secondary analyses. χ^2^ and Fisher's exact tests were used to compare discrete variables and a repeated measures linear mixed model including a time-treatment interaction was used to analyse neutrophil counts in follow-up. Differences in treatment effects between subgroups were assessed through the use of an interaction test (ie, a comparison of models with and without interaction terms). A prespecified exploratory aim was to determine the relationship between CD20 expression on acute lymphoblastic leukaemia blasts and response to rituximab treatment. The impact of CD20 expression on response to rituximab was analysed using an event-free survival model, which included an interaction with treatment group. We considered CD20 expression in two ways: calculated as a proportion and antigen density. Both were analysed as continuous variables and as split into tertiles, with the former also split at 20%, a cutoff used as positive in previous studies. We also assessed the role of CD20 calculated both ways in predicting events at 3 years using a time to event receiver operating characteristics (ROC) analysis allowing for censored event-free survival data.

Event-free survival and overall survival analyses were in the intention-to-treat population (all patients included, as randomly assigned, except misdiagnoses). Remission, MRD, and all safety analyses were limited to those who started trial treatment. All analyses were done in STATA (version 16.1). This study is registered with ClinicalTrials.gov as an International Standard Randomised Controlled Trial, NCT01085617.

### Role of the funding source

Neither the funder of the study (Cancer Research UK) nor Roche, who provided rituximab, had any role in study design, data collection, data analysis, data interpretation, or writing of the report

## Results

**Figure 1:**
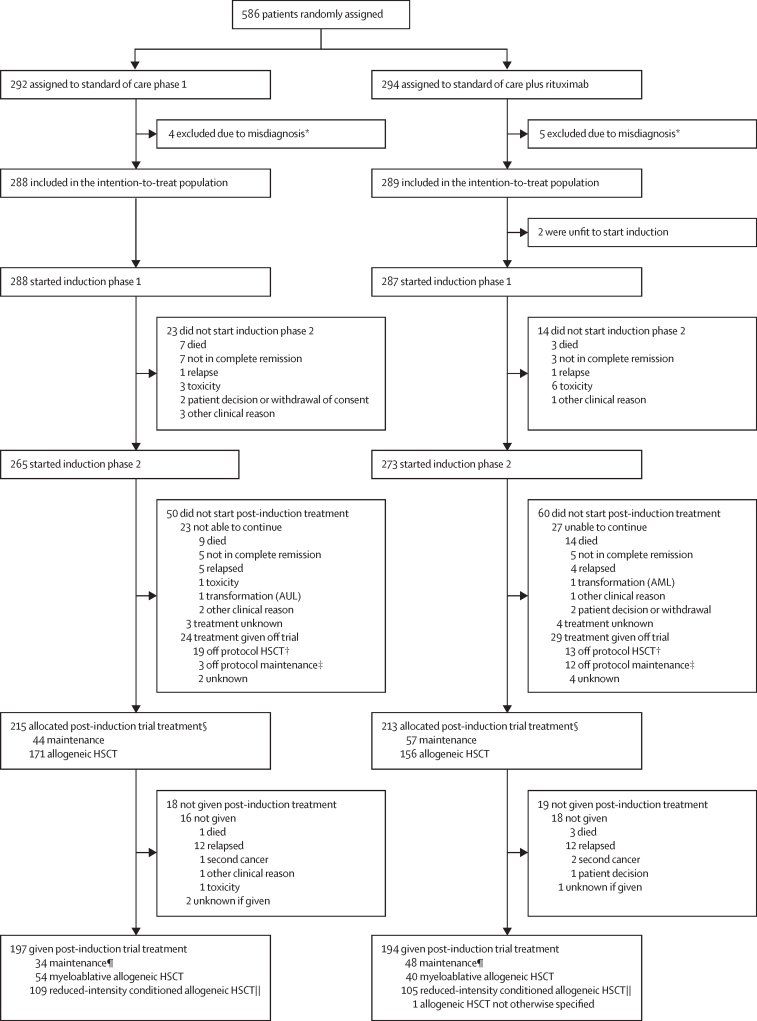
Trial profile AML=acute myeloid leukaemia. AUL=acute undifferentiated leukaemia. HSCT=haematopoietic stem-cell transplantation. POQR=positive outside quantifiable range. *Standard of care: biphenotypic leukaemia n=1, diffuse large B-cell lymphoma n=1, acute myeloid leukaemia n=1, Burkitt lymphoma n=1; standard of care plus rituximab: diffuse large B-cell lymphoma n=2, chronic myeloid leukaemia blast phase n=1, lymphoma not otherwise specified n=2. †Deviations from protocol conditioning or donor. ‡Deviation from trial maintenance pathway. §Minimal residual disease data availability for risk assessment: at the end of phase 1, 422 had a result (negative, positive, POQR, or indeterminate), 57 had specimen processed but no target was identified, 98 had insufficient or missing diagnostic specimen or end of phase 1 specimen not received or patient off trial; at the end of phase 2, 393 had a result (negative, positive, POQR, or indeterminate), 43 had specimen processed but no target was identified, 122 had insufficient or missing diagnostic specimen or end of phase 2 specimen not received or patient off trial. ∥Post-induction maintenance pathway includes intensification and four blocks of consolidation before the start of maintenance. ||Post-induction reduced intensity conditioned allogeneic HSCT pathway also includes intensification before HSCT.

Between April 19, 2012, and July 10, 2017, 586 patients were randomly assigned to a treatment group (standard of care n=292, standard of care plus rituximab n=294; figure 1). Of the 586 randomly assigned patients, nine were excluded due to misdiagnosis (figure 1). Thus, 577 patients were included in the intention-to-treat analysis, including two patients from the standard-of-care plus rituximab group who never started trial therapy. Table 1 shows the characteristics of patients who entered the study. Because teenagers and young adults up to 25 years of age were entered into the UK's paediatric study, the median age of UKALL14 participants was older than is typical in national study group adult acute lymphoblastic leukaemia trials (median age 45 years [IQR 35–54] in the standard-of-care group; 46 years [33–55] in the standard-of-care plus rituximab group; table 1).

**Table 1:** Baseline characteristics

			**Standard of care (n=288)**	**Standard of care plus rituximab (n=289)**	**Total (n=577)**
Age, years			45 (35–54; 22–65)	46 (33–55; 23–65)	46 (34–54; 22–65)
Age group					
	40 years or younger at random assignment		104 (36%)	108 (37%)	212 (37%)
	41 years or older at random assignment		184 (64%)	181 (63%)	365 (63%)
Sex					
	Male		159 (55%)	159 (55%)	318 (55%)
	Female		128 (44%)	130 (45%)	258 (45%)
	Intersex*		1 (<1%)	0	1 (<1%)
ECOG performance status					
	0–2		284 (99%)	283 (98%)	567 (98%)
	3–4		1 (<1%)	4 (1%)	5 (1%)
	Missing data		3 (1%)	2 (1%)	5 (1%)
Baseline white blood cell count, × 10^9^ cells per L			8·4 (0·1–583·1)	7·9 (0·4–889·6)	8·4 (0·1–583·1)
*BCR-ABL1* and breakpoint					
	*BCR-ABL1*-positive		86 (30%)	86 (30%)	172 (30%)
		p190	51 (18%)	55 (19%)	106 (18%)
		p210	29 (10%)	27 (9%)	56 (10%)
		Unknown breakpoint	6 (2%)	4 (1%)	10 (2%)
Any UKALL14 cytogenetic risk factor					
	Absent		101 (35%)	95 (33%)	196 (34%)
	Present		143 (50%)	140 (48%)	283 (49%)
	Missing data		44 (15%)	54 (19%)	98 (17%)
Genetic subtype					
	Complex karyotype		11 (4%)	6 (2%)	17 (3%)
	High hyperdiploid		6 (2%)	6 (2%)	12 (2%)
	JAK-STAT		15 (5%)	15 (5%)	30 (5%)
	*KMT2A* other		4 (1%)	2 (1%)	6 (1%)
	KMT2A-AFF1		17 (6%)	25 (9%)	42 (7%)
	Low hypodiploid or near-triploid		27 (9%)	22 (8%)	49 (9%)
	*TCF3-PBX1*		6 (2%)	8 (3%)	14 (2%)
	Other		81 (28%)	77 (27%)	158 (27%)
	Test failed or missing		35 (12%)	42 (15%)	77 (13%)
Baseline UKALL 14 risk†					
	Standard risk		36 (13%)	40 (14%)	76 (13%)
	High risk		241 (84%)	234 (81%)	475 (82%)
	Assumed standard risk‡		11 (4%)	15 (5%)	26 (5%)
Baseline UKALL14 risk excluding age					
	Standard risk		81 (28%)	83 (29%)	164 (28%)
	High risk		170 (59%)	158 (55%)	328 (57%)
	Assumed standard risk‡		37 (13%)	48 (17%)	85 (15%)

Data are median (IQR; range), median (IQR), or n (%). ECOG=Eastern Cooperative Oncology Group.

* Excluded from any analyses where sex is included.

† High presenting white blood cell count, high risk cytogenetics (*BCR-ABL1*-positive, *KMT2A/AFF1*, low hypodiploidy or near triploidy, complex [more than abnormalities]) or age 41 years and older.

‡ Patients with no high-risk features, but missing cytogenetic data (treated as standard risk).

Of the 287 patients in the standard-of-care plus rituximab group who began trial treatment, 274 (95%) received all four doses of rituximab; one (<1%) patient stopped rituximab due to an infusion-related reaction, five (2%) stopped rituximab due to toxicity of the chemotherapy protocol, one (<1%) missed a rituximab dose due to error, three (1%) stopped rituximab due to clinician choice, and three (1%) patients died before all doses could be administered (see appendix pp 2–4 for detailed data on dose reductions, delays, and omissions).

**Figure 2:**
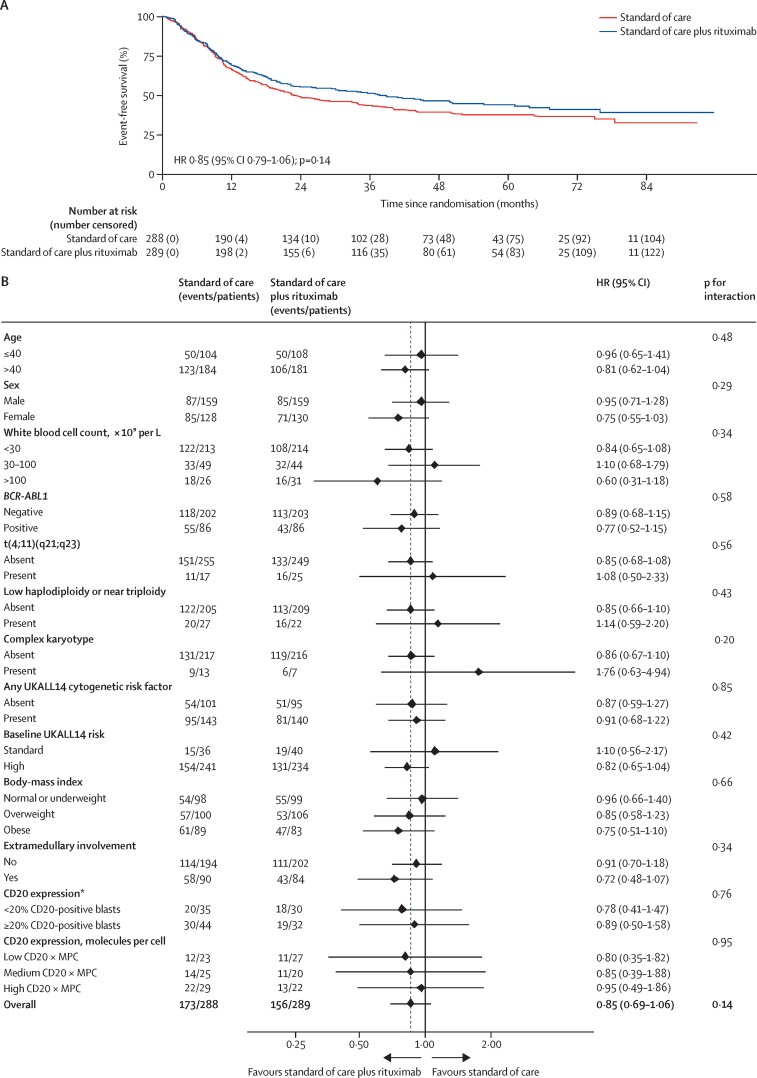
Event-free survival (A) and subgroup analysis of event-free survival (B) HR=hazard ratio. *GRAALL group cutoff.

After a median follow-up of 53·7 months (IQR 40·3–70·4), 179 patients had relapsed and 150 patients had died without relapse reported, providing 329 event-free survival events. Median event-free survival was 23·2 months (95% CI 19·2–34·0) in the standard-of-care group and 38·8 months (22·8–61·1) in the standard-of-care plus rituximab group. Event-free survival was not significantly different between the groups (HR 0·85 [95% CI 0·79–1·06]; p=0·14) with 3-year event-free survival rates of 43·7% (95% CI 37·8–49·5) for standard of care versus 51·4% (45·4–57·1) for standard of care plus rituximab (figure 2A, table 2).

**Table 2:** Efficacy data

		**Standard of care (n=288)**	**Standard of care plus rituximab (n=289)**	**p value**	**Univariable analysis, HR (95% CI); p value**	**Multivariable analysis, HR (95% CI); p value**	**Multivariable analysis including any transplantation, HR (95% CI); p value**
In remission by end of phase 1		256/288 (89%)	247/287 (86%)	0·44	..	..	..
In remission by end of phase 2		267/288 (93%)	272/287 (95%)	0·31	..	..	..
MRD-negative* remission end of phase 1		66/288 (23%)	77/287 (27%)	0·28	..	..	..
	Immunoglobulin heavy chain/T-cell receptor MRD quantification	0·00295 (1 × 10^−5^ to 0·15)	1 × 10^−5^ (1 × 10^−6^ to 0·0032)	0·040	..	..	..
	*BCR-ABL1* MRD value	0·20 (1 × 10^−5^ to 1·87)	0·091 (1 × 10^−5^ to 0·96)	0·59	..	..	..
MRD-negative* remission in induction		103 (36%)	100 (35%)	0·82	..	..	..
	Immunoglobulin heavy chain/T-cell receptor MRD quantification	1 × 10^−5^ (1 × 10^−6^ to 7 × 10^−4^)	1 × 10^−6^ (1 × 10^−6^ to 6·7 × 10^−4^)	0·35	..	..	..
	*BCR-ABL1* MRD value	1 × 10^−5^ (1 × 10^−5^ to 0·10)	1 × 10^−5^ (1 × 10^−5^ to 0·041)	0·54	..	..	..
Patients high risk by MRD alone		11 (<1%)	4 (<1%)	..	..	..	..
Event-free survival		..	..	..	0·85 (0·69 to 1·06); p=0·14†	0·88 (0·69 to 1·12); p=0·30	0·86 (0·68 to 1·09); p=0·22
	Number of events/number of patients	173/288	156/289	..	..	..	..
	3-year event-free survival (95% CI)	43·7% (37·8 to 49·5)	51·4% (45·4 to 57·1)	..	..	..	..
Deaths		..	..	..	0·88 (0·70 to 1·11); p=0·29	0·91 (0·70 to 1·18); p=0·46	0·89 (0·69 to 1·16); p=0·40
	Number of events/number of patients	148/288	134/289	..	..	..	..
	3-year overall survival (95% CI)	52·7% (46·6 to 58·4)	57·3% (51·3 to 62·8)	..	..	..	..
Relapses		..	..	..	0·85 (0·64 to 1·15); p=0·30	0·85 (0·61 to 1·18); p=0·34	0·84 (0·60 to 1·18); p=0·31
	Number of events/number of patients	92/267	83/272	..	..	..	..
	3-year relapse rate (95% CI)	31·0% (25·7 to 37·1)	26·3% (21·4 to 32·0)	..	..	..	..
Bone marrow relapses		..	..	..	0·88 (0·63 to 1·21); p=0·43	..	..
	Number of events/number of patients	77/267	70/272	..	..	..	..
	3-year bone marrow relapse rate (95% CI)	24·8% (20·0 to 30·6)	22·4% (17·8 to 28·9)	..	..	..	..
CNS relapses		..	..	..	0·73 (0·31 to 1·73); p=0·47	..	..
	Number of events/number of patients	12/267	9/272	..	..	..	..
	3-year CNS relapse rate (95% CI)	4·4% (2·4 to 7·7)	3·0% (1·5 to 6·0)	..	..	..	..
Non-relapse mortality		..	..	..	0·88 (0·62 to 1·26); p=0·49	0·94 (0·64 to 1·40); p=0·77	0·93 (0·63 to 1·39); p=0·74
	Number of events/number of patients	63/267	58/272	..	..	..	..
	3-year non-relapse mortality (95% CI)	23·7% (19·0 to 29·4)	20·6% (16·2 to 25·9)	..	..	..	..
Second malignancies‡		..	..	..	1·28 (0·64 to 2·58); p=0·48	..	..
	Number of events/number of patients	14/288	18/289	..	..	..	..
	3-year second malignancy rate (95% CI)	2·9% (1·5 to 5·8)	4·3% (2·5 to 7·5)	..	..	..	..
	Cumulative incidence of second malignancies at 5 years (95% CI)	6·3% (3·7 to 10·6)	7·6% (4·7 to 12·1)	..	..	..	..

Data are n/N (%), median (IQR), or n (%) except where otherwise stated. HR=hazard ratio. MRD=minimal residual disease.

* Includes positive outside the quantitative range, denominators for remission and MRD taken as the total numbers who started trial treatment, including those with missing remission or MRD data as no response. Excluding those who were in remission with missing or non-evaluable MRD gives rates at the end of phase 2 of 101 (55%) of 185 in the standard-of-care group and 100 (57%) of 175 in the standard-of-care plus rituximab group.

† HR 0·85 (95% CI 0·68–1·06); p=0·14 when adjusted for randomisation stratification factors.

‡ Only events occurring before relapse are reported, events were: acute myeloid leukaemia (standard of care n=2, standard of care plus rituximab n=6), acute undifferentiated leukaemia (standard of care n=1), lung (standard of care n=1, standard of care plus rituximab n=1), malignant histiocytosis (standard of care plus rituximab n=1), melanoma (standard of care n=2), Hodgkin lymphoma (standard of care n=1 [this patient also had melanoma]), myelodysplastic syndrome (standard of care n=2, standard of care plus rituximab n=2), neuroendocrine (standard of care plus rituximab n=1), non-melanoma skin cancer (standard of care n=1, standard of care plus rituximab n=2), oesophageal (standard of care n=1), post-transplantation lymphoproliferative disorder (standard of care n=2), renal (standard of care plus rituximab n=1), sarcoma (standard of care plus rituximab n=1), vestibular schwannoma (standard of care n=1), histiocytic sarcoma (standard of care plus rituximab n=1), myeloproliferative disorder (standard of care plus rituximab n=1), missing type (standard of care plus rituximab n=1).

Analyses of overall survival showed similar effects to event-free survival, with median survival times of 40·1 months (95% CI 33·2–75·0) for standard of care and 81·5 months (39·0–not reached) for standard of care plus rituximab (appendix p 21) and no significant difference between the groups (HR 0·88 [95% CI 0·70–1·11]; p=0·29). Time to relapse was analysable for the 539 patients who went into complete remission, with 181 events (table 2; and appendix p 22). The 3-year cumulative incidence of relapse was 31·1% (95% CI 25·8–37·1) for standard of care versus 26·3% (21·4–32·1) for standard of care plus rituximab (HR 0·85 [95% CI 0·63–1·15]; p=0·29). The addition of rituximab did not affect non-relapse mortality (table 2). Multivariable analyses, adjusting for important baseline factors were done for each of the survival endpoints (also shown in table 2) and results were consistent with the univariable analyses.

At the end of both phases of induction there was no significant difference between the groups in the proportion of patients in complete remission (table 2). There was also no significant difference in the numbers of patients in molecular remission (negative or POQR MRD) at the end of the first or second phase of induction (table 2). There was a significant difference in patient-specific immunoglobulin heavy chain/T-cell receptor MRD quantification at the end of phase 1 induction when analysed as a continuous variable, but the difference was not significant by the end of phase 2 induction, nor was there any significant difference in the patients whose MRD was assessed by *BCR-ABL1* quantification (table 2). Outcomes by *BCR-ABL1* status are shown in the appendix (pp 5, 21).

Table 3 shows the most common adverse events reported in UKALL14 induction (see appendix pp 7, 10, and 12–16 for detailed data for induction phase 1 and 2 adverse events). Grade 3–4 events were common, with infections and cytopenia being the most frequent events, but these events were similar in both treatment groups. There were 30 on-therapy mortality events noted during the reporting period (14 [5%] of 288 in the standard-of-care group, 16 [6%] of 287 in the standard-of-care plus rituximab group); the majority of these deaths were due to infections (eight [3%] in the standard-of-care group, 11 [4%] in the standard-of-care plus rituximab group) and in induction phase 1 and phase 2 (11 [4%] in the standard-of-care group, 13 [5%] in the standard-of-care plus rituximab group). There was no concern about long-term neutropenia in patients treated with rituximab, with no significant difference in neutropenia between the treatment groups (difference in absolute neutrophil count for standard of care plus rituximab *vs* standard of care 0·06 [95% CI –0·05 to 0·17]; p=0·31; repeated tests for patients in remission yearly after the end of therapy). 32 second malignancies were reported (table 2). We analysed the difference between treatment groups in all immune system adverse events, which included allergic, hypersensitivity, and anaphylaxis events. The overall rates of these events were expectedly low. There was no difference between the groups during the induction phases, but we did find significantly more immune system adverse events in the standard-of-care group than in the standard-of-care plus rituximab group (four [2%] of 185 *vs* 15 [8%] of 188; p=0·011) during the high dose methotrexate block, which includes two doses of pegylated asparaginase, consistent with our hypothesis that rituximab might reduce the incidence of pegylated asparaginase inactivation (appendix p 13).

**Table 3:** Most common adverse events in phase 1 induction

		**Standard-of-care group (n=286)**				**Standard-of-care plus rituximab group (n=284)**			
		Grade 1–2	Grade 3	Grade 4	Grade 5	Grade 1–2	Grade 3	Grade 4	Grade 5
Blood and lymphatic system disorders		71 (25%)	152 (53%)	..	0	77 (27%)	157 (55%)	..	0
	Anaemia	79 (28%)	138 (48%)	..	0	83 (29%)	145 (51%)	..	0
	Febrile neutropenia	..	41 (14%)	..	0	..	53 (19%)	..	0
Gastrointestinal disorders		196 (69%)	22 (8%)	..	0	186 (65%)	20 (7%)	..	0
	Constipation	98 (34%)	..	..	0	88 (31%)	..	..	0
	Diarrhoea	73 (26%)	..	..	0	64 (23%)	..	..	0
	Nausea	136 (48%)	..	..	0	133 (47%)	..	..	0
General disorders and administration site conditions		154 (54%)	..	..	1 (<1%)	148 (52%)	..	..	0
	Multi-organ failure	..	..	..	1 (<1%)	..	..	..	0
Infections and infestations		..	46 (16%)	..	3 (1%)	..	48 (17%)	..	2 (1%)
	Lung infection	..	..	..	1 (<1%)	..	..	..	0
	Sepsis	..	..	..	2 (1%)	..	..	..	2 (1%)
	Not otherwise specified bacterial infection	..	16 (6%)	..	0	..	15 (5%)	..	0
Investigations		..	45 (16%)	198 (69%)	1 (<1%)	..	41 (14%)	200 (70%)	0
	Alanine aminotransferase increased	..	27 (9%)	..	0	..	40 (14%)	..	0
	Alkaline phosphatase increased	..	15 (5%)	..	0	..	28 (10%)	..	0
	Blood bilirubin increased	..	21 (7%)	..	0	..	23 (8%)	15 (5%)	0
	Granulocytopenia	..	..	22 (8%)	0	..	..	20 (7%)	0
	Myelosuppression	..	..	..	0	..	..	22 (8%)	0
	Neutrophil count decreased	..	42 (15%)	179 (63%)	1 (<1%)	..	45 (16%)	181 (64%)	0
	Pancytopenia	..	..	20 (7%)	0	..	15 (5%)	23 (8%)	0
	Platelet count decreased	..	..	26 (9%)	0	..	..	21 (7%)	0
	White blood cell decreased	..	48 (17%)	121 (42%)	0	..	44 (15%)	140 (49%)	0
Metabolism and nutrition disorders		..	34 (12%)	..	1 (<1%)	58 (20%)	32 (11%)	..	0
	Acidosis	..	..	..	1 (<1%)	..	..	..	0
	Hyperglycaemia	..	..	..	0	..	15 (5%)	..	0
Nervous system disorders		125 (44%)	15 (5%)	..	1 (<1%)	130 (46%)	..	..	1 (<1%)
	Headache	96 (34%)	..	..	0	98 (35%)	..	..	0
	Intracranial haemorrhage	..	..	..	1 (<1%)	..	..	..	1 (<1%)
Skin and subcutaneous tissue disorders		105 (37%)	..	..	0	105 (37%)	..	..	0
	Alopecia	60 (21%)	..	..	0	61 (21%)	..	..	0
Any adverse events		..	54 (19%)	200 (70%)	5 (2%)	..	50 (18%)	205 (72%)	3 (1%)

Events presented: all grade 5 events, grade 3–4 events occurring in 5% or more patients, and grade 1–2 events occurring in 20% or more patients. Patients with more than one event are counted once in the system organ class row but are shown in multiple individual adverse event type rows; if a grade 3 or 4 event occurred in less 10% of patients or a grade 1–2 event occurred in less than 15% of patients that event type is not listed but is included in the total events for that system organ class. See appendix pp 7–10 for full details of all grade 3–4 events and grade 1–2 adverse events occurring in 10% or more patients.

Preplanned subgroup analyses examined treatment effects by *BCR-ABL1* status and by CD20 expression levels. The induction and survival outcomes by *BCR-ABL1* status are shown in appendix p 5 and Kaplan–Meier curves for event-free survival and overall survival by *BCR-ABL1* status are shown in appendix p 21. There was no significant difference in treatment effect for any outcome in these subgroup analyses.

Central laboratory eight-colour flow cytometry using cryopreserved diagnostic cells was possible in 150 patients (79 in the standard-of-care group, 71 in the standard-of-care plus rituximab group). Patients with stored cells available were more likely than patients without stored cells to have high presenting white blood cell counts (72 [48%] of 150 *vs* 78 [18%] of 427; p<0·0001) or have high-risk cytogenetics (93 [67%] of 138 *vs* 190 [56%] of 341; p=0·019), possibly explained by a larger number of cells available at diagnosis for these patients. Exposure to dexamethasone in vitro significantly upregulated CD20 on acute lymphoblastic leukaemia cells from six of eight patient specimens tested (appendix p 23). Results from the analysis of the role of CD20 expression as a prognostic factor are shown in the appendix (p 14). Analysed as a continuous variable, we found that a 1 log increase in CD20 expression was associated with a 23% increase in risk of an event (HR 1·23 [95% CI 1·05–1·44]; p=0·012). Event-free survival was shorter among patients with 20% or more blasts expressing CD20 (76 [51%] of 150) compared with patients with less than 20% blasts expressing CD20 (74 [49%]); however, this difference was not statistically significant: HR 1·44 (95% CI 0·95–2·21; p=0·087; appendix p 14). CD20 antigen density, calculated as molecules per cell × blast proportion was also clearly and significantly related to outcome, although it did not appear to add a greater level of discrimination to proportion of blasts expressing CD20 (ROC area under the curve for prediction of a 3-year event-free survival event: 0·60 for CD20 antigen density and 0·62 for percentage of blasts expressing CD20). CD20 expression, as quantified either by proportion of blasts expressing CD20 or CD20 molecules per cell, remained significantly associated with inferior event-free survival when adjusted for the other baseline prognostic factors (appendix p 14). CD20 expression was analysed as a continuous variable by using 20% cutoff and by splitting into tertiles. There was no evidence that greater CD20 expression improved the efficacy of rituximab (appendix p 14).

A preplanned subgroup analysis of event-free survival is shown figure 2B. There were no significant interactions with any of the main risk-stratification factors including age, cytogenetic risk group, presenting white blood cell count, or risk status overall. The same was the case for subgroup analyses of overall survival, relapse rate, and non-relapse mortality (data not shown).

To assess the effect of allogeneic HSCT and any interaction with randomised allocation, time-to-event outcomes were analysed in a multivariable model including allogenic HSCT as a time varying covariate. When adjusted for baseline risk factors, allogeneic HSCT appeared to be beneficial (appendix pp 15, 24). Although the risk of non-relapse mortality increased, this appeared to be balanced out by improvements in time to relapse, leading to overall improvements in both event-free survival and overall survival (appendix pp 15, 24). There were no significant interactions found between rituximab and allogeneic HSCT overall, although the effect of allogeneic HSCT on non-relapse mortality was reduced when rituximab was given (HR 1·57 [95% CI 0·82–2·98] *vs* 2·16 [1·15–4·06]; interaction p=0·16). The effect of transplantation on time-to-event outcomes is shown in detail in the appendix (p 15).

The effect of rituximab in patients who received reduced-intensity conditioned allogeneic HSCT as postremission therapy was similar to that seen in the whole population (event-free survival HR 0·86 [95% CI 0·60–1·24]; p=0·42, overall survival HR 0·79 [0·53–1·18]; p=0·29, relapse rate HR 1·05 [0·65–1·70]; p=0·85, non-relapse mortality HR 0·70 [0·40–1·23]; p=0·21). However, those who received myeloablative conditioned allogeneic HSCT had a significantly greater event-free survival if they were in the standard-of-care plus rituximab group versus the standard-of-care group (3-year event-free survival 50·5% [95% CI 36·2–63·2] *vs* 71·0% [53·5–82·9]; HR 0·47 (95% CI 0·23–0·95]; p=0·032; appendix p 24). Similar results were seen for overall survival (HR 0·49 [95% CI 0·23–1·07]; p=0·066), relapse rate (HR 0·47 [0·15–1·47]; p=0·18), and non-relapse mortality (HR 0·47 [0·15–1·47]; p=0·18), suggesting that there might have been both a reduction in release rate and non-relapse mortality driving the apparent benefit in the standard-of-care plus rituximab group followed by myeloablative conditioned allogeneic HSCT (appendix p 24). There was no obvious trend in causes of death between the two treatment groups (data not shown), nor any difference in rates of GVHD. To attempt an intention-to-treat analysis, we generated a larger cohort in which we included all patients younger than 41 years with high-risk acute lymphoblastic leukaemia at the end of induction, or a sibling donor, who could have received myeloablative conditioned allogeneic HSCT. In this cohort of 136 patients (standard of care n=74, standard of care plus rituximab n=62), the effect of standard of care plus rituximab was not as great as in the main analysis (3-year event-free survival 51·7% [95% CI 39·4–62·7] in the standard-of-care group *vs* 62·9% [49·1–74·0] in the standard-of-care plus rituximab group; HR 0·67 [95% CI 0·40–1·11]; p=0·12; appendix p 24). There was no difference between standard of care and standard of care plus rituximab in the cumulative incidence of GVHD for patients receiving myeloablative conditioned allogeneic HSCT (3-year cumulative incidence 72·2% [95% CI 60·0–83·3] *vs* 73·2% [58·8–85·9]; HR 1·12 [95% CI 0·69–1·81]; p=0·65). The incidence of any GVHD in patients receiving reduced-intensity conditioned allogeneic HSCT was also not different between the two treatment groups (3-year cumulative incidence 65·3% [95% CI 56·3–74·1] *vs* 52·4% [43·2–62·2]; HR 0·75 [95% CI 0·53–1·07]; p=0·12).

Factors of significance on multivariable analysis were largely as expected; increasing age, higher presenting white blood cell count, high-risk cytogenetics (*KMT2A/AFF1*, low hypodiploidy or near triploidy, complex [>5 abnormalities]), and CD20 expression (where analysable) were all significant prognostic factors for inferior event-free survival (appendix p 17). *BCR-AB1*-positive acute lymphoblastic leukaemia was not an adverse risk factor in this analysis. High-risk cytogenetics portended inferior overall survival (HR 1·56 [95% CI 1·13–2·17]; p=0·0013) with a similar trend seen for higher presenting white blood cell count (HR 1·08 [1·00–1·18]; p=0·055). Both factors were significantly associated with a higher relapse rate (HR 1·54 [95% CI 1·01–2·35]; p=0·031 and HR 1·17 [1·05–1·30]; p=0·0034; appendix p 21). Older age was the only factor significantly impacting non-relapse mortality (HR for a 10-year increase 1·31 [95% CI 1·09–1·58]; p=0·0039).

## Discussion

Four doses of rituximab added to standard-of-care chemotherapy during induction treatment for all adults aged 25–65 years, regardless of CD20 expression or *BCR-ABL1* status, gave an event-free survival HR of 0·85 (95% CI 0·69–1·06), which was greater than the 0·71 the study was powered to detect and hence did not meet the primary endpoint. However, we found a possibly clinically beneficial 8% improvement in event-free survival at 3 years and a 15 month longer median event-free survival in the investigational group. The benefit we observed appears to result from a reduction in relapse rate, as opposed to a better early response. Although there was a modest, non-significant reduction in the first timepoint MRD level in the standard-of-care plus rituximab group, MRD responses were equivalent by the main assessment timepoint. There was a similar magnitude of difference in overall survival (HR 0·88 [95% CI 0·70–1·11]) with median overall survival of 40·1 months (95% CI 33·2–75·0) for standard of care compared with 81·5 months (39·0–not reached) with standard of care plus rituximab. 3-year overall survival was 52·7% (95% CI 46·6–58·4) with standard of care compared with 57·3% (51·3–62·8) for standard of care plus rituximab, mirroring the event-free survival advantage, and again a non-significant difference, of adding rituximab. Adding rituximab to induction therapy was safe—we did not detect any increase in short-term adverse events in the standard-of-care plus rituximab group, nor any issues with long-term neutropenia, and there was no difference in non-relapse mortality or numbers of on-therapy mortality (grade 5) events between the groups**.** By contrast to the MD Anderson single-arm study,^1^ we did not find any relationship between toxicity and age. We believe that our results are generalisable across patients with acute lymphoblastic leukaemia because of our broad eligibility criteria and the fact that our trial was open in all UK centres that treat adult acute lymphoblastic leukaemia. On the basis of national incidence data, we estimate that we recruited approximately 80% of the incident population.

The most relevant comparable study is the randomised study conducted by the GRAALL group,^2^ which enrolled a cohort with *BCR-ABL1*-negative acute lymphoblastic leukaemia and with CD20 expression on more than 20% of blasts—the median age was 6 years younger than in our present study. Our study found no evidence of a differential effect of rituximab in relation to age, and although interaction tests did not show significance, treatment effects were generally larger for *BCR-ABL*-positive patients for both event-free survival and time to relapse, and there was a significant difference in bone marrow relapses between *BCR-ABL*-positive and *BCR-ABL*-negative patients, suggesting that these population differences did not account for the more modest benefit of rituximab that we observed overall.

We were able to quantify CD20 expression in a central laboratory in a subset of patients, by availability of cryopreserved cells. As well as central evaluation of the proportion of cells expressing CD20, we also precisely quantified the number of CD20 molecules per cell, affording two methods by which to analyse the role of CD20 expression. Because we enrolled patients regardless of CD20 expression, we expected the full range of CD20 values would provide a robust analysis of the relationship of rituximab response with CD20 expression. The fact that only a subset of patients had cryopreserved cells available is a clear limitation. However, in this group we found no evidence of an interaction between benefit from rituximab and CD20 expression, suggesting that any benefit from rituximab is unrelated to the traditional cutoff of 20% blasts expressing CD20. However, the crucial difference that most likely explains the stronger, statistically significant finding of benefit in the GRAALL study is the difference in the number of doses of rituximab administered, a total of 16 doses across the protocol, compared with the four doses given during induction only in the present study. Although it cannot be proven that the benefit from 16 doses of rituximab in the GRAALL study could extend to those with lower levels of CD20-expressing blasts or those with *BCR-ABL*-positive disease, our data suggest there could be a benefit of adding rituximab to the treatment of all patients with B-cell acute lymphoblastic leukaemia, regardless of the proportion of blasts expressing CD20 or *BCR-ABL1* status.

Despite only a subset of patients contributing to the analysis of CD20 expression, the number included was sufficient to detect a large and significant effect of CD20 level on prognosis, with higher levels of expression associated with inferior event-free survival even when adjusted for other baseline risk factors, including cytogenetics, age, and white blood cell count. Our data also suggest that more precise calculation of CD20 molecules per cell does not add anything to the simpler and widely available standard flow cytometric method of quantifying the proportion of cells expressing CD20. When the prognostic effect of CD20 was analysed including allogeneic HSCT as a time-varying covariate, we found some evidence of an interaction, suggesting a larger effect of CD20 expression on prognosis in those not transplanted. These data support the conclusion from Bachanova and colleagues^10^ that allogeneic HSCT might overcome the poor prognostic relevance of CD20 expression. The binary cutoff of 20% blasts expressing CD20 does not have any biological basis and has not been chosen as the point that has the best balance of sensitivity and specificity to predict outcome. If CD20 expression is to be used to direct rituximab therapy in patients with acute lymphoblastic leukaemia, a more clinically meaningful cutoff than 20% expression should be chosen.

The precise mechanism whereby rituximab adds event-free survival and overall survival benefit in patients with acute lymphoblastic leukaemia is not clear, but there are several plausible candidate mechanisms, all of which might co-exist. Direct mechanisms of action such as signalling-induced cell death, complement-dependent cytotoxicity, and antibody dependent cellular cytotoxicity are likely to play a role, as seen in other blood cancers.^16^ The mechanisms that depend on cellular cytotoxicity might be less readily activated if the drug is given largely during myelosuppression, as done here. This suggestion might further explain the requirement for an extended dosing schedule. Synergy with other elements of acute lymphoblastic leukaemia therapy might provide indirect mechanisms through which rituximab plays a role in B-cell acute lymphoblastic leukaemia. In our study, the role of pegylated asparaginase was evaluated in a single arm manner for all study participants, with an exploratory aim to determine whether there was a relationship between rituximab and asparaginase activity or anti-asparaginase antibody generation. Those data are not fully available and will be presented with the results of the asparaginase question; however, our immune system adverse events data add some evidence in favour of a potential synergy. Of interest, the GRAALL data showed fewer allergic reactions to native, *Escherichia coli* asparaginase.

We were also interested in any effect of rituximab on allogeneic HSCT outcomes and preplanned this analysis. Peripheral blood B-cell depletion persists for some months after rituximab treatment, providing a plausible biological relationship. We were surprised that only patients who received myeloablative conditioned allogeneic HSCT had a significantly greater event-free survival if treated with standard of care plus rituximab with an HR of 0·47 (95% CI 0·23–0·95); p=0·032. We could not find a single explanation—both relapse rate and non-relapse mortality were reduced by the same magnitude and there was no apparent difference in GVHD rates. This effect was not seen for patients who received reduced-intensity conditioned allogeneic HSCT. We have attempted to assess whether there was any interaction between treatment and the use of allogeneic HSCT with an analysis incorporating HSCT as a time varying covariate. Although there are limitations to this analysis (transplanted patients differing in risk and earlier response to treatment), we found no evidence of an interaction or evidence that the addition of rituximab is harmful in a transplanted population.

Limitations of this trial are both specific, including the choice of only four doses of study drug and an incomplete specimen collection for correlation with CD20 levels, and general to studies of acute lymphoblastic leukaemia. General issues include strong relationships between patient age and outcome, the practical problem of evaluating novel agents when added to long and complex standard-of-care therapy, and the difficulties of analysing outcome data in a disease where many patients are assigned, via adaptive risk stratification, to allogeneic HSCT as part of their treatment. The length of time needed to conduct studies in uncommon diseases is also a concern.

In summary, the UKALL14 B-cell randomisation in which four doses of rituximab were added to standard of care did not show a statistically significant improvement in event-free survival. The full benefit of rituximab in patients with acute lymphoblastic leukaemia is likely to require more prolonged administration of rituximab, as demonstrated by the GRAALL randomised controlled trial.

## Data sharing

There was no data sharing plan set out at the start of this study. Specific requests for non-identifiable data for valid academic reasons as judged by the trial management group will be granted, with appropriate data sharing agreement, and should be addressed to the chief investigator (AKF).

## Declaration of interests

AKF received institutional peer-reviewed grant funding from Cancer Research UK, the Medical Research Council, and Servier; received consulting fees from Amgen; has a leadership/fiduciary role for the British Society of Haematology (unpaid); and is on a data safety and monitoring board for Novartis. AAK has received honoraria from Kite and Gilead; has received institutional peer-reviewed grant funding from Millennium pharmaceutics, Bristol Myers Squibb, Amgen, Celgene, Merck Sharp and Dohme, Janssen-Cilag, Pfizer, and Cancer Research UK; and is on a data safety and monitoring board or advisory committee for the University of Birmingham. AKM has received honoraria form Amgen, Roche, Abbvie, and Celgene; has received institutional peer-reviewed grant funding from Janssen and AstraZeneca; has received meeting support from Roche, Celgene, and Abbvie; and is on a data safety and monitoring board or advisory committee for Novartis. AVM has received institutional peer-reviewed grant funding from Cancer Research UK, Blood Cancer UK, the EU Innovative Medicines Initiative, and North East Children's Cancer Fund; has a leadership/fiduciary role for the ALLTogther Consortium Board and the Genetics Committee and Science Committee (unpaid); and has received honoraria from Amgen. AZC has received honoraria from Pfizer, Amgen, and Hartley Taylor; and is on a data safety and monitoring board or advisory committee for Pfizer. BB has received institutional peer-reviewed grant funding from Novartis/Haematology Society of Australia and New Zealand (New Investigator Scholarship). CJR has received honoraria from Kite; is on a data safety and monitoring board or advisory committee for Incyte, Gilead, and Pfizer; and has a leadership/fiduciary role as the National Cancer Research Institute Adult ALL Group Chair (unpaid). DIM has received consulting fees from Amgen, Pfizer, Kite, and Novartis; and honoraria from Amgen, Pfizer, and Kite. DY has received meetings support from Amgen, Servier, and Jazz; and is on a data safety and monitoring board or advisory committee for the University of Birmingham, Gilead, and Pfizer. KEB has received research grant support from Blood Cancer UK. LC-H has received institutional peer-reviewed grant funding from Millennium pharmaceutics, Bristol Myers Squibb, Amgen, Celgene., Merck Sharp and Dohme, Janssen-Cilag, Pfizer, and Cancer Research UK. MC has received institutional peer-reviewed grant funding from Cure Leukaemia, Blood Cancer UK, Cyclacel, and Incyte; has received consulting fees from Pfizer, Novartis, and Jazz; has received payment or honoraria from Pfizer, Novartis, Jazz, and Astellias; and has received meeting support from Novartis. PS has received institutional peer-reviewed grant funding from Cancer Research UK, Blood Cancer UK, and Servier. RK has received honoraria from Amgen and Pfizer; and is on a data safety and monitoring board or advisory committee for Amgen. TFM has received honoraria from Amgen, Pfizer, Kite, Gilead, Janssen, Roche, Servier, Novartis, and Celgene; has received institutional peer-reviewed grant funding from Janssen and AstraZeneca; and has received meeting support from Novartis, Amgen, Pfizer, Kite, Gilead, Celgene, Daiichi Sankyo, and Atara. All other authors declare no competing interests.
